# Population Structure in *Naegleria fowleri* as Revealed by Microsatellite Markers

**DOI:** 10.1371/journal.pone.0152434

**Published:** 2016-04-01

**Authors:** Bénédicte Coupat-Goutaland, Estelle Régoudis, Matthieu Besseyrias, Angélique Mularoni, Marie Binet, Pascaline Herbelin, Michel Pélandakis

**Affiliations:** 1 Univ Lyon, Université Lyon 1, CNRS UMR 5240 Microbiology Adaptation and Pathogenesis, Villeurbanne, France; 2 Univ Lyon, Université Lyon 1, Villeurbanne, France; 3 Univ Lyon, Université Lyon 1, ISPB EA 4446 Bioactive Molecules and Medicinal Chemistry, Lyon, France; 4 EDF Research and Development, Laboratoire National d’Hydraulique et Environnement, Chatou, France; National Cheng-Kung University, TAIWAN

## Abstract

*Naegleria* sp. is a free living amoeba belonging to the Heterolobosea class. Over 40 species of *Naegleria* were identified and recovered worldwide in different habitats such as swimming pools, freshwater lakes, soil or dust. Among them, *N*. *fowleri*, is a human pathogen responsible for primary amoeboic meningoencephalitis (PAM). Around 300 cases were reported in 40 years worldwide but PAM is a fatal disease of the central nervous system with only 5% survival of infected patients. Since both pathogenic and non pathogenic species were encountered in the environment, detection and dispersal mode are crucial points in the fight against this pathogenic agent. Previous studies on identification and genotyping of *N*. *fowleri* strains were focused on RAPD analysis and on ITS sequencing and identified 5 variants: euro-american, south pacific, widespread, cattenom and chooz. Microsatellites are powerful markers in population genetics with broad spectrum of applications (such as paternity test, fingerprinting, genetic mapping or genetic structure analysis). They are characterized by a high degree of length polymorphism. The aim of this study was to genotype *N*. *fowleri* strains using microsatellites markers in order to track this population and to better understand its evolution. Six microsatellite loci and 47 strains from different geographical origins were used for this analysis. The microsatellite markers revealed a level of discrimination higher than any other marker used until now, enabling the identification of seven genetic groups, included in the five main genetic groups based on the previous RAPD and ITS analyses. This analysis also allowed us to go further in identifying private alleles highlighting intra-group variability. A better identification of the *N*. *fowleri* isolates could be done with this type of analysis and could allow a better tracking of the clinical and environmental *N*. *fowleri* strains.

## Introduction

The free living-amoeboflagellate *Naegleria fowleri* is the causative agent of primary amoebic meningoencephalitis (PAM) in humans, a rare but rapidly fatal disease of the central nervous system. The species is thermophilic and is found in soil and aquatic environments. Most of the fatal infections due to *N*. *fowleri* happen in young people exposed to warm water in ponds, swimming pools and lakes [1]. Two other *Naegleria* species are thermophilic and share the same habitat as *N*. *fowleri*: *Naegleria australiensis* which is pathogenic in mice and *Naegleria lovaniensis* which is harmless. Another species, *Naegleria italica* was reported to be pathogenic in mice but was rarely isolated [2,3].

Due to the lack of morphological characters within the genus *Naegleria*, the identification of *N*. *fowleri* was carried out by various techniques using immunological and molecular tools with specific PCR primers [4–11]. Molecular markers allowed to investigate intra-species variability which was first observed by using RFLP [12,13], and then by using RAPD markers displaying a higher level of diversity [14,15]. Therefore, the RAPD markers evidenced five main variants, namely South Pacific (SP), Chooz (CHO), Cattenom (CAT), Widespread (WP), and Euro-American (EA) which are not always correlated with geographical origin. The existence of RAPD variants was confirmed by the analysis of the 5.8S rRNA gene and the internal transcribed spacers (ITS), except for the CAT variant that could not be differentiated, since it is identical to the SP variant [6,16–19].

ITS analyses remain very useful for typing, but are not sufficiently informative for population genetic studies, or for a thorough tracking of *N*. *fowleri* strains. In this respect, genetic markers such as microsatellites are more suitable. Microsatellites are tandemly repeated short motifs of 1 to 6 base pairs and are widely distributed in the prokaryotic and eukaryotic genomes. They display variations in the number of repeats and exhibit a high level of polymorphism. Microsatellites are powerful markers for population genetics with broad spectrum of applications and thus can be used for biodiversity, fingerprinting, population studies, genetic mapping within populations, clonal structure and evolutionary history [20,21]. Therefore, microsatellites are of great interest in the study of eukaryotic genetic diversity, in particular for protozoa such as *Plasmodium* sp. [22,23], *Trypanosoma cruzi* [24], *Cryptosporidium parvum* [25] or *Leishmania* sp. [26,27].

In this study, we describe and examine six microsatellites in the different variants of *N*. *fowleri* which were initially identified by RAPD and ITS. Our objective was to improve the understanding of population structure of this pathogenic species, to enhance the typing for a better tracking, and to better understand their dispersal. The analysis of *N*. *fowleri* strains with these microsatellites provided valuable new information about their population structure.

## Materials and Methods

### Sampling

The 47 *N*. *fowleri* strains and DNA analyzed in this study are shown in Table 1. The French strains, which were isolated between 2009 and 2012, originated from different geographical sites. The strains with identical first letters in the nomenclature came from the same French site. The genotype of the strains were identified and confirmed by sequencing the ITS region (Table 1). Representatives of the variants were cloned.

**Table 1:** DNA and strains used with geographical origin.

Name	Geographical origin	RAPD/ITS variant^a^	Source^b^	Type^c^	Year of isolation	Reference
Kul	Belgium	EA/3	H	S	1973	[14]
Moj200	France	EA/3	E	S	1987	[14]
WM	USA	EA/3	H	D	1969	[15]
SW1	USA	EA/3	E	D	1976	[15]
EPA-1911s	USA	EA/3	E	D	-	[6]
Lovell	USA	EA/3	E	D	1974	[14]
NG060	Australia	SP/5	E	D	-	[6]
PAa	New-Zealand	SP/5	E	D	1972	[14]
Northcott	Australia	SP/5	H	D	1971	[14]
CA66	Australia	SP/5	H	D	1966	[14]
PA117	Australia	SP/5	E	D	1972	[15]
MW4U	Australia	SP/5	E	D	1972	[15]
Mst	Australia	SP/5	H	D	1974	[14]
Na420c	France	CAT/5	E	S	1988	[14]
J26(50)45E	Japan	CAT/5	E	D	1990	[15]
PLC-2	Mexico	WP/2	H	D	1990	[15]
Enterprise	USA	WP/2	E	D	1976	[15]
124	USA	WP/2	E	D	1984	[15]
A1	France	EA/3	E	S	2010	This study
A2	France	EA/3	E	S	2010	This study
A3	France	EA/3	E	S	2010	This study
A4	France	EA/3	E	S	2009	This study
B1	France	EA/3	E	S	2011	This study
B2	France	EA/3	E	S	2011	This study
B3	France	EA/3	E	S	2011	This study
B4	France	EA/3	E	S	2012	This study
B5	France	EA/3	E	S	2012	This study
C1	France	WP/2	E	S	2011	This study
C2	France	WP/2	E	S	2011	This study
C3	France	WP/2	E	S	2011	This study
C4	France	WP/2	E	S	2011	This study
C5	France	WP/2	E	S	2011	This study
D1	France	EA/3	E	S	2010	This study
D2	France	EA/3	E	S	2010	This study
E1	France	CAT/5	E	S	2010	This study
E2	France	CHO/4	E	S	2012	This study
E3	France	CHO/4	E	S	2012	This study
F1	France	WP/2	E	S	2010	This study
F2	France	WP/2	E	S	2012	This study
F3	France	WP/2	E	S	2012	This study
F4	France	WP/2	E	S	2012	This study
F5	France	WP/2	E	S	2012	This study
F6	France	WP/2	E	S	2012	This study
G1	France	WP/2	E	S	2010	This study
G2	France	WP/2	E	S	2011	This study
G3	France	WP/2	E	S	2011	This study
G4	France	WP/2	E	S	2011	This study

^a^ EA: Euro-american, SP: South pacific; WP: Widespread; CAT; Cattenom, CHO, chooz variants from [15]; the number of the ITS genotype from [19];

^b^ H: human or E: environmental isolates;

^c^ D: DNA or S: strains.

### Culture and DNA extraction

All strains were cultured on non nutrient agar (NNA) plates with *E*. *coli* cells layers and incubated at 30°C for 4 to 7 days. Isolated strains were recovered from NNA plates and scrapped in order to extract DNA. Extraction was performed with Nucleospin Tissue kit (Macherey Nagel, Hoerdt, France) according to the manufacturer's protocol. DNA quantity and quality, for both RNA and protein contaminations, were controlled with a Nanodrop 1000 spectrophotometer. For PCR analysis, all DNAs were diluted to a final concentration of 10 ng/μL.

### Microsatellite isolation

#### Partial genomic librairies

Total DNA was extracted by phenol-chloroform extraction according to the conventional procedure. To avoid the presence of extrachromosomal DNA, total DNA was loaded onto a 0.8% agarose gel (SeaPlaque Agarose, Lonza-Ozyme, Montigny-Le-Bretonneux, France) and run for 22h in TAE buffer at 20V and 16mA. The selected DNA corresponding to chromosomal DNA was digested with Sau3A (Boehringer Mannheim—Roche Diagnostics, Meylan, France). Restriction fragments between approximately 300 to 900 bp were transferred onto DEAE paper (Sanbrook 1989). The fragments were ligated within PUC18 vector (Amersham biosciences—GE healthcare, Glattbrugg, Switzerland) and amplified after transformation into competent XL blue cells (Amersham biosciences—GE healthcare).

#### Screening and sequencing

Clones were transferred on solid LB medium plates and transferred onto Hybond-N Nylon membrane (Amersham biosciences—GE healthcare). The screening was performed with an equal mix of oligonucleotides (TC)_10_, (TG)_10_, (CAC)_5_CA, CT(CCT)_5_, CT(ATCT)_6_, and (TGTA)_6_TG, labelled with the DIG oligonucleotide tailing kit (Boehringer Mannheim—Roche Diagnostics). Positive clones were directly analyzed by sequencing (Amersham, Pharmacia-Biotech T7 sequencing kit—GE healthcare). Five of these clones were retained for the analysis.

### Microsatellites PCR amplification and analysis with capillary sequencer

The five clones were amplified with different probes labeled with FAM fluorochrome (Table 2). For further analyses, NG42 and NG141 were amplified into 2 separate fragments (NG42-1, NG42-2 and NG141-3, NG141-5, respectively). Amplifications were performed with EconoTaq (Lucigen, Middletown, USA) using an initial denaturation step at 95°C for 5 min, followed by 40 cycles at 95°C for 10s, 57°C (or 50°C) for 20s and 72°c for 20s and a final elongation step at 72°C for 7 min. Fluorescent amplified fragments were analyzed on a ABI 3730 capillary sequencer with a GeneScan 600 LIZ size standard (Applied biosystems—Life Technology, Saint Aubin, France) from the Genomics platform of Nantes (Biogenouest Genomics). Results were analyzed with Peakscanner software (Applied biosystems).

**Table 2:** Microsatellites loci and primers.

Locus	Microsatellite^a^	Allele size range (bp)	Number of alleles	Primers U/L^b^
NG25	(GT)_8_	110–116	4	AATATTGCTGATGCGAAGGG/AATTGCACCACCAACTCCG
NG42-1	(CT)_5_…(ACC)_7_	220–226	4	GCAGCACTCACTCCTCCTC/CACCTCTCCCTCTTACAACAG
NG42-2	(GGT)_7_…(GGT)_4_	222–252	5	GATTTCCTGCTGGACGATGA/CCCAAGTCGTCCATGATGAGA
NG69	(TTC)_7_…(CAT)_6_G(GGT)_6_…(CTT)_6_	238–245	3	CAACCTGTTCCCAAGATTTGTAAG/TATGGATAAGGAAAGAAGTGATACAAG
NG141-3	(TG)_6_…(TG)_5_…(TG)_4_…(GT)_6_…(GT)_6_	162–216	10	CTCAATGTAGAAAATGCTAAT/TGGAATGAATGGAATATACTC
NG141-5	(GT)_11_	153–163	5	GAGAGTATATTCCATTCATTCCA /TTGATCATCCTCATCATTCCACC

^a^ repetitions of microsatellite are indicated for KUL strain.

^b^ U: Upper primer (5'-3') and L: lower primer 5'-3'.

### Cloning and sequencing of microsatellite alleles

Different alleles were cloned in pCR2.1 TOPO TA cloning vector (Invitrogen—Life Technologies) and plasmids were extracted with QIAprep Spin Miniprep Kit (Qiagen, Courtaboeuf, France), according to the manufacturer's protocol. Amplifications using M13R and M13F primers were performed with EconoTaq after an initial denaturation step of 95°C for 5 min, followed by 40 cycles of 95°C for 10s, 55°C for 20s and 72°c for 20s and a final elongation of 72°C for 7 min. ABI 3730XL sequencer was used by Genoscreen (Lille, France) to sequence alleles.

### Data analyses

Genepop 4.2.2 software [28] was used to calculate allele frequency-based correlation (F_IS_, F_ST_ and F_IT_). F_IS_ is the proportion of the total inbreeding within a population due to inbreeding within sub-populations. It ranges between -1 and 1, where a negative value corresponds to an excess of heterozygotes, and a positive value to heterozygote deficiency. F_IS_ = 0 indicates Hardy-Weinberg allele proportions. F_ST_ is the proportion of the total inbreeding in a population due to differentiation among sub-populations. Mean F_ST_ estimates over loci in each population were calculated with the FSTAT software (version 2.9.3.2, [29]) using Nei estimators. F_IT_ is the total inbreeding in a population due to both inbreeding within sub-populations, and differentiation among sub-populations. Expected and observed heterozygosity (He and Ho, respectively) were estimated with R software using Adegenet package [30].

Factorial correspondence analysis (FCA) implemented in GENETIX 4.05 software [31] was performed, which places the individuals in a three-dimensional space according to the degree of their allelic state similarities.

Assignment testing was performed by using the GENECLASS 2.0 software package [32]. The Bayesian method of Rannala and Mountain [33] was selected to perform self-assignment tests with algorithm of Paetkau et al. [34] on a simulation with 1000 individuals and alpha = 0.001.

Matrix of genetic distances was performed with the POPULATIONS 1.2.32 software and the Cavalli-Sforza and Edwards model. Phylogenetic networks were inferred from the distance matrix obtained from the microsatellite dataset by using the Neighbor-Net method in SplitsTree 4.13.1 [35].

### Ethics Statement

The French sites (A-G) mentioned in Materials & Methods and Table 1 are industrial sites for which the authorizations have been obtained. These sites are secure and their precise location cannot be mentioned. The field studies did not involve endangered or protected species.

## Results

Among the partial library, 3500 clones were screened and 30 were positives and were sequenced. A number of clones were excluded because, either no microsatellites were present, or because amplification was not possible, probably due to chimeric alleles. Five clones were selected for intra-specific analysis and referred to as NG25, NG42, NG69, NG115 and NG141. NG42 and NG141 loci contained different microsatellite repetitions and were divided in two parts for further analysis (NG42-1 and NG42-2 and NG141-3 and NG141-5, respectively). NG115 was found to be monomorphic with one allele at 115 bp for all strains and was not used thereafter. Thus, a total of 6 microsatellites markers were used for this analysis. The locus composition is indicated in Table 2 and shows that most of them are imperfect with repeats.

Microsatellite analyses displayed one or two alleles and can be considered as homozygous or heterozygous. The exception came from NG69 displaying three alleles for most of the strains, due to the presence of a duplication of this locus (Table 3). Therefore, one identical 214 bp allele was present in all samples. It was subsequently cloned and its sequence was identical to the other ones from the NG69 locus. The duplication was confirmed by the blast analysis of the *N*. *fowleri* genome recently sequenced [36], which indicates that each locus is represented on one contig, except for NG69, which is present on two different contigs.

**Table 3:** Allelic combination of the microsatellite markers for the strains examined.

ITS variant	Strain or Origin	NG25			NG42-1				NG42-2					NG69^a^				NG141-3										NG141-5					Total number of alleles
		110	114	116	220	223	225	226	222	225	226	228	252	214	238	241	245	162	164	167	179	180	183	185	197	212	216	153	155	157	159	163	
**Euro-American**																																	**13**
	Kul		X			X				X				X	X	X					X								X				
	Moj		X			X				X				X	X	X					X							X					
	D1		X			X				X				X	X	X					X	X							X				
	D2		X			X				X				X	X	X					X				X				X				
	B1-5		X			X				X			X	X	X	X					X	X							X				
	A1-4		X			X				X				X	X	X					X	X							X				
	SW1		X			X				X				X	X	X		X							X				X				
	WM		X			X	X			X				X	X	X					X				X				X				
	EPA1911		X			X				X				X	X	X					X								X				
	Lovell		X			X				X				X	X	X					X				X				X				
**South Pacific**																																	**16**
	NG060					X				X				X	X	X			X												X		
	PAa	X				X		X		X	X			X		X				X						X					X		
	PA117		X			X				X				X	X							X					X		X		X		
	Northcott		X			X				X				X	X							X					X		X		X		
	CA66		X			X			ND					ND								X					X		X		X		
	Mst	ND			ND					X	X			ND						X						X				X	X		
	MW7U		X			X				X				X	X												X				X		
**Widespread**																																	**12**
	G1-4			X		X				X		X		X	X	X			X			X							X	X			
	C1-5			X		X				X		X		X	X	X			X			X							X	X			
	F1-6		X			X				X		X		X	X	X			X						X				X	X			
	124			X	ND					X		X		X	X	X			X			X						ND					
	Enterprise			X	ND					X		X		X	X	X			X			X						ND					
	PLC2			X		X				X		X		X	X	X			X			X							X	X			
**Chooz**																																	**9**
	E2-3			X		X				X				X	X	X							X	X					X			X	
**Cattenom**																																	**11**
	E1		X		X					X				X		X			X				X						X	X			
	Na420c		X		X	X				X				X		X			X				X						X	X			
	JP45E		X			X			X	X				X		X	X		X				X						X				

ND: not determined.

^a^ Since the 214 pb allele is present in all strains, it was not consider in the analysis of the data.

### Genetic diversity

The tested microsatellites revealed a different level of polymorphism in our samples. The NG141-3 was the most variable marker with 10 alleles identified, whereas the other microsatellites produced between 3 to 5 alleles.

The highest number of alleles was 16 and was found for the SP strains whereas strains from CAT and those from EA and WP presented 11, 13 and 12 different alleles, respectively (Table 3).

For the NG141, NG69 and NG42-2 markers, the overall observed heterozygosity per locus was higher than expected and reflected an excess of heterozygosity which was confirmed by the negative F_IS_ values (Table 4). In contrast, the observed heterozygosity per locus was lower than expected for the NG42-1 marker, and displayed a deficiency of heterozygosity. Surprisingly, no heterozygous were found with the NG25 microsatellite, therefore explaining the values of 0 and 1 obtained with H_o_ and F_IS_, respectively.

**Table 4:** Genetic characteristics of the microsatellite markers observed in the populations of *N*. *fowleri*.

	H_o_	H_e_	F_ST_	F_IS_	F_IT_	D_ST_
NG25	0.000	0.4527	0.5782	1.000	1.000	0.309
NG42-1	0.0454	0.0875	0.4174	0.2302	0.5515	0.060
NG42-2	0.5454	0.4384	0.2183	-0.4883	-0.1633	0.061
NG69	0.8666	0.5108	0.0847	-0.7993	-0.6468	0.068
NG141-3	0.8936	0.8100	0.2915	-0.4113	0.0000	0.223
NG141-5	0.6818	0.6242	0.3462	-0.4923	0.0244	0.186
**All**			0.312	-0.358	0.066	0.151

H_o_, observed heterozygosity; H_e_, expected heterozygosity; F_ST_, inbreeding coefficient among individuals within populations; F_IS_, inbreeding coefficient within the population; F_IT_, inbreeding coefficient as the global population; D_ST_, genetic diversity.

### Population structure

**Fig 1:**
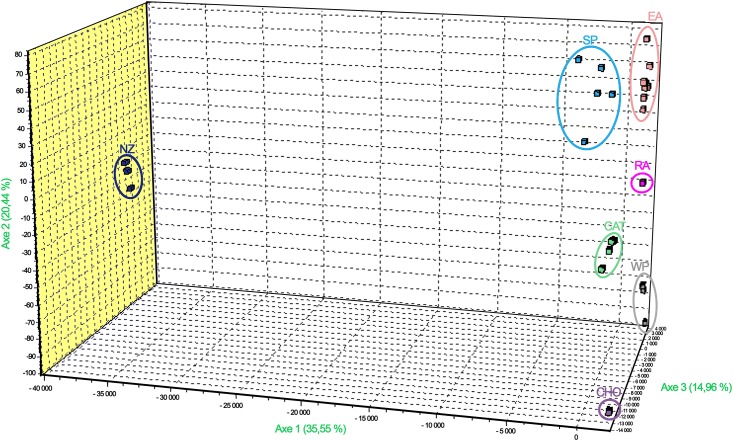
Factorial correspondence analysis (FCA) of *Naegleria fowleri* strains. The strains labelled in blue, orange, pink, green, grey, purple and black correspond to those that were assigned to the SP, EA, RA, CAT, WP, CHO and NZ respectively.

The factorial correspondence analysis (FCA) showed that we recovered the 5 variants previously identified, EA, WP, SP, CHO and CAT with two additional ones: NZ and RA, respectively included in the SP and WP groups based on the previous RAPD and ITS analysis (Fig 1). Identified by microsatellite markers, these variants will be named as genetic groups. The GENECLASS program was performed to assess the assignment of the 47 strains to these seven genetic groups. 97.9% of the strains were correctly assigned. Indeed, the SP strain NG060 and the CAT strain JP45E were not assigned. The EA strain SW1 was placed within the WP with a very weak probability (p = 0.016) (S1 Table).

**Fig 2:**
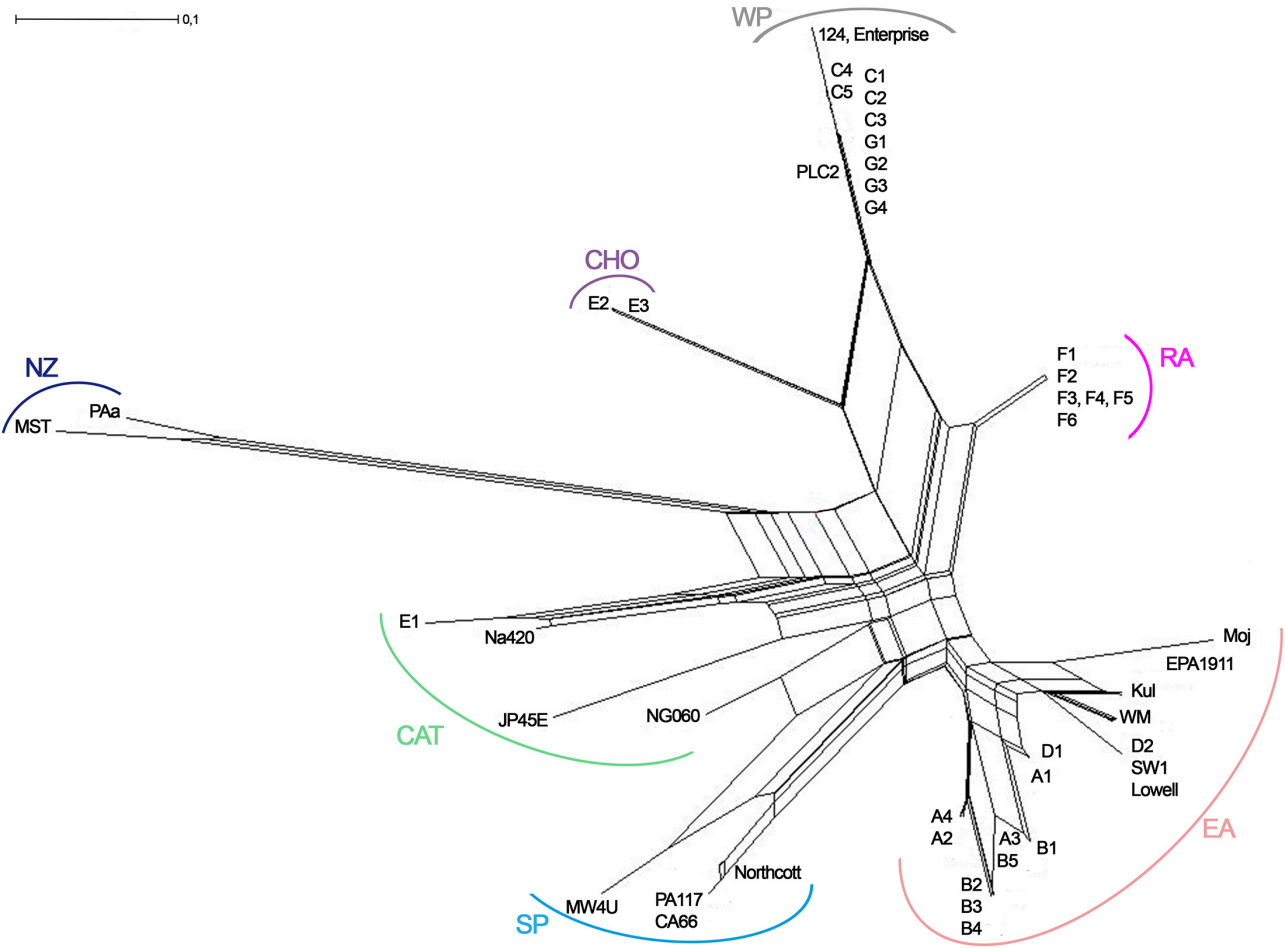
The NeighborNet network of the seven genetic groups of *N*. *fowleri* identified by the microsatellite markers. The strains labelled in blue, orange, pink, green, grey, purple and black correspond to those that were assigned to the SP, EA, RA, CAT, WP, CHO and NZ respectively.

The existence of this structure was also supported by the NeighborNet network (Fig 2). The genetic differentiation between the seven groups was very high with F_ST_ values varying from 0.29 to 0.61 (Table 5).

**Table 5:** Pairwise comparison of Fst between the seven genetic groups.

	EA	SP	NZ	WP	RA	CHO	CAT
EA	0.0000*						
SP	0.3145*	0.0000					
NZ	0.5956*	0.6117	0.0000				
WP	0.4740*	0.5144*	0.5738	0.0000			
RA	0.2883*	0.3773	0.5746	0.3846*	0.0000		
CHO	0.4674	0.5432	0.6111	0.3415	0.5161	0.0000	
CAT	0.3334*	0.4455*	0.4380	0.4797*	0.2906	0.4423	0.0000

*indicates significant difference between group pairs (p value < 0.05).

FCA as well as the NeighborNet network included the three strains SW1, JP45E and NG060 into their respective groups. The FCA showed that the NZ group is very apart from the other ones whereas the SP and EA groups were found to be close. With the NeighborNet network, the SP, NZ and CAT groups are closely related to the EA group. CHO and RA groups are branching with WP (Fig 2). However, the ambiguous splits throughout the network indicate that the relationships within the clusters and groups are not resolved.

### Variation within genetic groups

The different groups displayed specific profiles with NG141-3 marker (Table 3). Every marker was able to identify some genetic groups, but not all. For example, NG42-2 specifically detected the genetic WP and SP groups but EA, CHO and CAT groups shared the same alleles. Additionally, differentiation within a given group could be observed. Therefore, the NG141-3 locus generated four different profiles within the EA group. The heterozygous profile (179 bp-180 bp) was only observed in France, the heterozygous profile (162 bp-197 bp) was only observed in the USA, and the two remaining ones (179 pb and 179 bp-197 bp) were both found in USA and France (Table 3). Note that the heterozygous profile 179 bp-197 bp was found only once in a French site among the twenty French strains examined, whereas the heterozygous profile 179 bp-180bp was recovered on the majority of them.

A number of strains also had a specific microsatellite profile with one or several loci. Therefore, the B strains displayed a singular profile with the 252 bp allele at the NG42-2 locus. This private allele was not found elsewhere. The difference of 27 bp between this allele and the other one (225 bp) led us to sequence them. As expected, the result revealed an insertion of 27 nucleotides that is not due to microsatellite variations. This insertion, located at the beginning of the sequence could suggest the mismatch of the upper primer that could therefore explain the weaker intensity peak for the 252 bp allele compared to the 225 bp allele (S1 Fig). Other private alleles could be identified, particularly in the SP group (Table 3). The two strains KUL and Moj 200 shared a same homozygous profile of 179 bp for the NG141-3 locus that was not observed elsewhere either. For the same DNA strains, similar profile results were observed, even though some parameters were changed, such as Taq DNA polymerases (Econotaq, Ozyme and HotMaster Taq), PCR programs (temperature and number of cycles), or sequencers (MEGABACE 1000 or 96-capillary ABI 3730xl DNA Analyzer) (S2 Table).

## Discussion

Microsatellite markers revealed a higher level of discrimination than any other markers used until now, and were used in this study to further explore the genetic diversity of *N*. *fowleri* and establish its population structure. To observe the same microsatellite profiles within a few years apart with different material and procedure i.e. different DNA extraction kits and sequencers, demonstrates the reproducibility of these markers. Another important point is the stability of the allelic profiles. Indeed, the DNA strains isolated a long time ago show the same profiles as recently isolated. Particulary, identical specific profiles with NG42 and NG141 were observed for the B or F strains.

In agreement with previous studies, numerous heterozygous were observed with most of the microsatellite markers suggesting that this species is diploid [37–39]. This was confirmed by the genome of *N*. *fowleri* recently published [36]. All loci are present in one copy in the *N*. *fowleri* genome except for NG69 that is present on two contigs. This confirms our duplication hypothesis that explains the third allele of 214 bp present in all samples.

The previous analyses based on the RAPD and ITS data identified five main genetic groups EA, WP, SP, CAT, and CHO. The two last groups encompass predominantly French strains and exhibited similarities with SP [6,15]. Specifically, the strains from the CHO group are exclusively French, originating in eastern France, and they belong to type 4 [15, 19]. While the CAT group contains both French and Japanese strains. The microsatellite markers confirmed these five groups and within two groups (SP and WP) enabled the identification of two new groups, i.e., the NZ group which was considered in the previous analysis based on the RAPD and ITS data as a member of SP and RA which belonged to the WP group. Each of these groups emerged from the different genetic analyses such as factorial correspondence analysis and NeighborNet network in SplitsTree. Furthermore, the different groups were found to be well differentiated as given by the very high F_ST_ value.

The examination of the NeighborNet network could provide some information about the evolutionary history of this species. The branching pattern of SP, NZ and CAT as well as EA was in agreement with the results obtained with RAPD and ITS data [6,15]. In contrast, the branching position of CHO with the WP was unexpected since CHO displays ITS and RAPD similarities with SP, NZ and CAT [6,15]. According to the microsatellite data, the CHO and RA groups shared the same specific profile with the NG25 locus (116 bp), and could explain this grouping. Another unexpected result as to do with the emergence of the additional group RA. On the basis of the RAPD and ITS data, this group belonged to the WP group. However, in this study, it can be considered as a distinct group according to the F_ST_ value (Table 5 and Figs 1 and 2). In any cases, the RA group remains very closely related to the WP group but more extensive sampling is needed to confirm the branching pattern obtained in this study.

As previously found with RAPD and ITS analyses, ubiquist genotypes were also observed with the microsatellite markers, yet found to be more discriminating. This confirmed that a significant variability was not necessarily correlated with the geographical origin of the strains. For example, within the EA group, the two French strains, D1 and D2 from the same geographical site displayed two different allele profiles, and one of which was identical to the USA strains (WM and Lovell). Similar cases were observed within the WP group. Conversely, within the CAT group, the Japanese and French strains are distinguishable by using microsatellites in contrast to the RAPD and ITS markers. Additionally, the South Pacific strains exhibited allelic profiles that were not found elsewhere, and showed higher variability than the other groups.

The ubiquity of the genetic groups suggested clonal dispersion of the species. Another point which underlined this type of propagation is the excess of heterozygosity produced from most markers reflected by the negative F_IS_ value. However, no heterozygosity was observed with NG25 although variability was present (four alleles described).

Most of the variants EA, WP and SP were observed both from clinical and environmental isolates (Table 1). So far, the other variants CAT and CHO are only of environmental origin. As already mentioned, the fact that some variants are either less prevalent in the environment or endemic could explain why they are not currently found in clinical cases [40]. So it is very likely that all variants of *N*. *fowleri* are pathogens. Additionally, no degree of virulence between them has been reported so far.

As a potential diagnostic tool, tracking *N*. *fowleri* is important to understand the dispersal mode and the colonization of this species in the various sites. In a previous article, we underlined the interest to fingerprint the pathogenic species [6]. Discriminating markers allow better exploration of the environment and to further establish the level of genetic diversity in the sites. In addition, since more and more PAM cases are reported worldwide [41], these markers could allow a better identification of the *N*. *fowleri* isolates in patients. Until now, the ITS markers were currently used for detecting the variability of the *N*. *fowleri* species. However the genotypes which were identified are not sufficiently informative. For example, the EA group, which was only represented by the ITS genotype 3 is widely distributed in the European and American continents. In contrast, at least four different profiles, were obtained within the EA group with our microsatellite markers. This enables to better understand and identify the clinical and environmental *N*. *fowleri* strains.

### Conclusion

Microsatellite markers confirmed the genetic heterogeneity within the species and can lead to a better tracking of the *N*. *fowleri* isolates. These markers were able to differentiate the genetic groups and within these, some strains by highlighting the private alleles.

Additionally, microsatellite markers allowed to better understand the evolutionary history of this species as well as its dispersal mode by determining the prevalence of the genetic groups in the sites. Our results exhibited the four similar genetic groups previously identified with ITS and RAPD studies. However, the different clustering methods as well as all the tests used in this study produced two supplementary genetic groups, RA and NZ, that were previously included in the WP and SP groups, respectively. Interestingly, the RA group is very closely related to WP. Previous results based on ITS data showed that all members of WP and RA were identical. One might then suggest that WP and RA have diverged from a same lineage. However, additional data should be collected to confirm if it is indeed an isolated group. Similarly, other geographical strains such as the African and Asian ones are necessary to confirm the genetic groups, and additional microsatellite markers could be identified from the recent sequencing of the *N*. *fowleri* genome in order to reinforce this study.

## Supporting Information

S1 Fig Alignment sequence of the 2 alleles (225bp and 252bp) of the NG42 locus. (DOCX)

S1 Table Assignment of the 47 *N*. *fowleri* strains with GENECLASS. (XLSX)

S2 Table N. fowleri analysis. *N*. *fowleri* strains examined before this study.
(XLSX)
