# Supplementary material for: Population Structure in Naegleria fowleri as Revealed by Microsatellite Markers
Source: PLoS One. 2016 Apr 1;11(4):e0152434. doi: 10.1371/journal.pone.0152434 (PMC4818093; doi:10.1371/journal.pone.0152434)
Supplement: S1 Fig — (DOCX) [file pone.0152434.s001.docx]

**Sequence alignment**

NG42_225bp gatTTCCTGCTGGACGATGAAATTCTTTTC---------------------------AAA

NG42_252bp GATTTCCTGCTGGACGATGAAATTCTTKWTTTCMTKGTGGRMGRWGRARKTSTTKTMAWA

NG42_225bp ACTGTGGGAGGAGGAGGTGTTGTACTCTTTTGCAACAACACACGTTGTGTTTCCGAGGTG

NG42_252bp AYTGTSRGVRGAGGAGGTGTTGTACTCTTTTGCAACAACACACGTTGTGTTTCCGAGGTG

NG42_225bp GTGGTGGTGGTGGTGGTGGCGGAGGAGGAAGTGGTGGTGGTGGTTGGAGAAGCAGCAGCA

NG42_252bp GTGGTGGTGGTGGTGGTGGCGGAGGAGGAAGTGGTGGTGGTGGtTGGAGAAGCAGCAGCA

NG42_225bp GCACTCACTCCTCCTCTCTGAAGTAGAATTCTACTTGGCGAATCATCATTCAGTCTTCTC

NG42_252bp GCACTCACTCCTCCTCTCTGAAGTAGAATTCTACTTGGCGAATCATCATTCAGTCTTCTC

NG42_225bp ATCATGGACGACTTGGGA

NG42_252bp ATCNTGGACGACTTGGG-
